# *Corydalis sunhangii* (Papaveraceae): A new species from Xizang, China, based on plastome and morphological data

**DOI:** 10.1002/ece3.11225

**Published:** 2024-04-04

**Authors:** Jun‐Tong Chen, Magnus Lidén, Xian‐Han Huang, Shun‐Quan Yang, Xin‐Jian Zhang, Qun Liu, Qi‐Lun Su, Guo‐Jun Hua, Jian Luo, Tao Deng

**Affiliations:** ^1^ State Key Laboratory of Plant Diversity and Specialty Crops Kunming Institute of Botany, Chinese Academy of Sciences Kunming Yunnan China; ^2^ Systematic Biology, EBC Uppsala University Uppsala Sweden; ^3^ School of Life Sciences Yunnan Normal University Kunming Yunnan China; ^4^ Association for Nature and Science Popularization of Xihu District Zhejiang China; ^5^ Institute of Plateau Ecology Tibet Agricultural and Animal Husbandry University Nyingchi Xizang China

**Keywords:** China, *Corydalis*, new species, phylogeny, plastome

## Abstract

A new species of Papaveraceae, *Corydalis sunhangii*, in the section *Trachycarpae*, is described and illustrated from Nyingchi City, Xizang, China. The new species has some resemblance to *Corydalis kingdonis*, but differs by radical leaves prominent, usually several, blade tripinnate (vs. insignificant, few, blade bi‐ to triternate); cauline leaf usually one, much smaller than radical leaves, usually situated in lower half of stem (vs. usually two, larger than radical leaves, concentrated in upper third of stem); racemes densely 13–35‐flowered (vs. rather lax, 4–11‐flowered); claw of lower petal shallowly saccate (vs. very prominently and deeply saccate); capsule oblong, with raised lines of dense papillae (vs. broadly obovoid, smooth). Phylogenetic analysis, based on 68 protein‐coding plastid genes of 49 samples, shows that *C. sunhangii* is not closely related to any hitherto described species, which is consistent with our morphological analysis.

## Graphical abstract

In this paper, a new species of Papaveraceae, *Corydalis sunhangii*, in the section *Trachycarpae*, is described and illustrated from Nyingchi City, Xizang, China. Integrating detailed morphological data and molecular evidence, we believe that these evidences satisfy the required diagnostic criteria to identify *C. sunhangii* as a new good species.

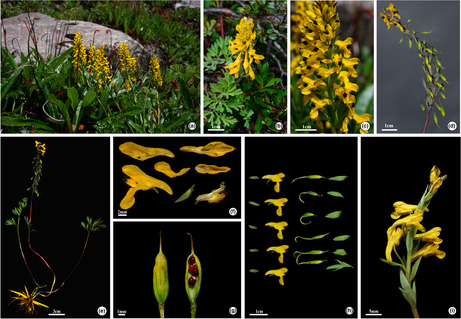

## 1 INTRODUCTION

The genus *Corydalis* DC. in Lamarck & De Candolle (1805: 637), the largest genus of Papaveraceae, contains ca. 530 species (Chen et al., 2023) mainly distributed in the North Temperate zone with the highest diversity in China (Wu et al., 1999; Zhang et al., 2008). The name *Corydalis* has a very checkered history. Linnaeus (1737, 1753) treated all species of the subfamily Fumarioideae in a single genus *Fumaria*. His original 11 species are now distributed among nine different genera. The first modern user of the generic name *Corydalis* was Medikus (1789), picking up an old name from Dillenius (1719), for a South African genus. However, this genus had already been named *Cysticapnos* by Miller (1754). Then Ventenat (1803) used *Corydalis* to refer to the species now called *Capnoides sempervirens* (L.) Borkh. and *Adlumia fungosa* (Aiton) Britton. During the 19th century, *Corydalis* came to refer to any Fumarioideae with zygomorphic flowers and many‐seeded capsules. However, according to the principle of priority, *Corydalis* would be a synonym of *Cysticapnos*, and *Corydalis* in the modern sense should be called *Pistolochia* (Bernhardi, 1800). If the genus name *Pistolochia* was used, it will inevitably cause many name changes. To stabilize the situation, *Corydalis* was therefore proposed for conservation by Mansfeld (1953). He, however, unfortunately chose the sole species of *Capnoides* Mill. (*C. sempervirens*) as type for the conserved name, thereby making things even worse. This was eventually rectified (Lidén, 1981) to allow for continuation of the established use of *Corydalis*, with the conserved type *C. solida* (L.) DC. And Lidén (1981) proposed *Corydalis* DC. in Lamarck & De Candolle (De Candolle, 1805) as the initial document of *Corydalis*.

Previous molecular analyses, based on few molecular markers and incomplete taxonomic sampling, were clearly inadequate to delimit sections and subgenera (Lidén et al., 1995, 1997; Wang, 2006; Xu et al., 2022). Recently, Chen et al. (2023) proposed a new classification of *Corydalis* with four subgenera and 39 sections, based on 65 protein‐coding plastid genes and 152 universal low‐copy nuclear genes, using samples covering all previously recognized sections and independent “series.”

China has a vast territory with a wide range of complex and diverse topographies and soils and covering several climate types (Chen et al., 2020), which contribute to the high species diversity of *Corydalis*. The Himalaya‐Hengduan Mountains region is the diversity center of *Corydalis* (Wang, 2006). Recently, we discovered a species of *Corydalis*, collected from Sejila Mountain in Nyingchi City, which did not agree with any previously known species. Located in southeast Xizang, west of Nanga Bawa Peak, Sejila Mountain is a watershed between the Niyang River and the Palong Zangbo River, and the highest elevation of Sejila Mountain is about 5300 m (Luo et al., 2021).

Our new species belongs to *Corydalis* sect. *Trachycarpae* (Fedde) Fedde, characterized by fasciculate fleshy storage roots, reflexed fruiting pedicels, and corolla often with distinct dark veins. The section name *Trachycarpae* also has a checkered history. This name as no formal rank stated “Gruppe” was first proposed by Fedde (1924). Subsequently, sect. *Trachycarpae* was officially established by Fedde (1936), who selected *C. trachycarpa* Maxim. as type for this section. However, this section name was often used as synonym of sect. *Fasciculatae* (Lidén, 1986; Wu et al., 1999). In 1889, Maximowicz established sect. *Fasciculatae* Maxim. and in sect. *Fasciculatae*, *C. curviflora* Maxim. as well as from the remotely related sect. *Trachycarpae* (e.g. *C. trachycarpa*) were included (Maximowicz, 1889). However, he did not designate a type. The first publication in which a type is designated for sect. *Fasciculatae* is Lidén (1986), who selected *C. cashmeriana* Royle (an earlier described species, which is referred to by Maximowicz as closely related to *C. curviflora*) as “type,” which amounts to an “inadvertent lectotypification” according to §7.11 of the Code (Turland et al., 2018). However, Wu et al. (1999), instead designated *C. trachycarpa* as “type” for sect. *Fasciculatae*, presumably unaware of the earlier action by Lidén. And Wu et al. (1999) used sect. *Fasciculatae* as official name for this section and sect. *Trachycarpae* as a synonym for this section, and recorded 17 species and five varieties, which belong to two different sections according to Chen et al. (2023). However, Zhang et al. (2008) adopted sect. *Trachycarpae* as the official name for this section group and recorded 52 species. From then on, sect. *Trachycarpae* as official name was stable and widely accepted. Afterward, there were three new species published in this section (Lidén et al., 2013; Pathak et al., 2013). *Corydalis dorjii* D.G.Long and *C. lupinoides* C.Marquand & Airy Shaw previously included within sect. *Trachycarpae* in Zhang et al. (2008) were removed from this section and belong to sect. *Lupinoides* J.T. Chen, T. Deng, Lidén & H. Sun in Chen et al. (2023). *Corydalis* milarepa was wrongly placed in sect. *Chrysocapnos* Wendelbo in Zhang et al. (2008) for the type specimen had no roots, and these species were moved into this section in Chen et al. (2023). According to Chen et al. (2023), there are 55 species in this section, of which 52 species occur in China, including 47 endemics. It is one of three sections in *Corydalis* that have over 50 species (Chen et al., 2023). Our new species is not closely similar to any other known species, and it is described below as *Corydalis sunhangii* J.T. Chen, T. Deng & M. Lidén.

## 2 MATERIALS AND METHODS

### 2.1 Morphological comparison

Specimens of *Corydalis sunhangii* were collected from Nyingchi City in Xizang and studied at the herbarium of KUN, UPS, and HNWP. Morphological characters, recorded for the new species, were based on dried specimens and photographs.

### 2.2 Phylogenetic reconstruction

Our molecular analysis was performed based on 49 samples (including 5 newly sequenced) from 41 species and represents the most comprehensive phylogeny of sect. *Trachycarpae* so far. *Corydalis inopinata* Prain ex Fedde, *C. pseudodrakeana* Lidén, and *C. lupinoides* were chosen as outgroups, based on results of Chen et al. (2023). Vouchers are deposited in the herbaria KUN and PE. Vouchers information for new sequences are presented in Table 1.

**TABLE 1:** Vouchers of five newly sequenced *Corydalis.*

Order	Code name	Species	Voucher specimen
1	C151	*C. sunhangii*	LiuJQ‐08XZ‐074 (KUN)
2	C412	*C. weisiensis*	01458 (PE)
3	C503	*C. sunhangii*	QTP‐LuoJ‐1768 (KUN)
4	C521	*C. bijiangensis*	GLGS‐496 (KUN)
5	C524	*C. mayae*	GLGS‐572 (KUN)

Based on Chen et al. (2023), we chose 68 shared protein‐coding plastid genes to perform the phylogenetic analysis. All sequences were obtained from the genome skimming data. DNA extraction, library preparation, and sequencing were conducted at Novogene (Beijing, China). The sequencing reads were assembled using GetOrganelle v1.7.4.1 (Jin et al., 2020). All linear chloroplast genomes were annotated using PGA (Qu et al., 2019). The plastid genes were aligned using MAFFT version 7.475 (Katoh & Standley, 2013), followed by minor manual corrections. Gaps were treated as missing data.

Phylogenetic relationships were assessed using Bayesian Inference (BI) analyses and maximum likelihood (ML). The ML phylogenetic tree was conducted in the CIPRES webserver (http://www.phylo.org) using RAxML version 8.2.12 (Stamatakis, 2014). Substitution model options were set to auto, followed by 1000 bootstrap replicates. The best‐fit model TVM + I + G estimated in jModelTest (Posada, 2008) using Bayesian information criterion (BIC) for Bayesian inference (BI) analysis. BI tree was conducted by MrBayes version 3.2.7 (Ronquist & Huelsenbeck, 2003) using the settings: Bayesian trees were started from random trees; four Markov Chain Monte Carlo (MCMC) simulations were run simultaneously and sampled every 1000 generations for a total 2 million. Runs were considered to have converged to stationarity when their average standard deviation of split frequencies was less than 0.01 and the first 25% trees were discarded as burn‐in.

## 3 RESULTS

### 3.1 Morphology

**FIGURE 1:**
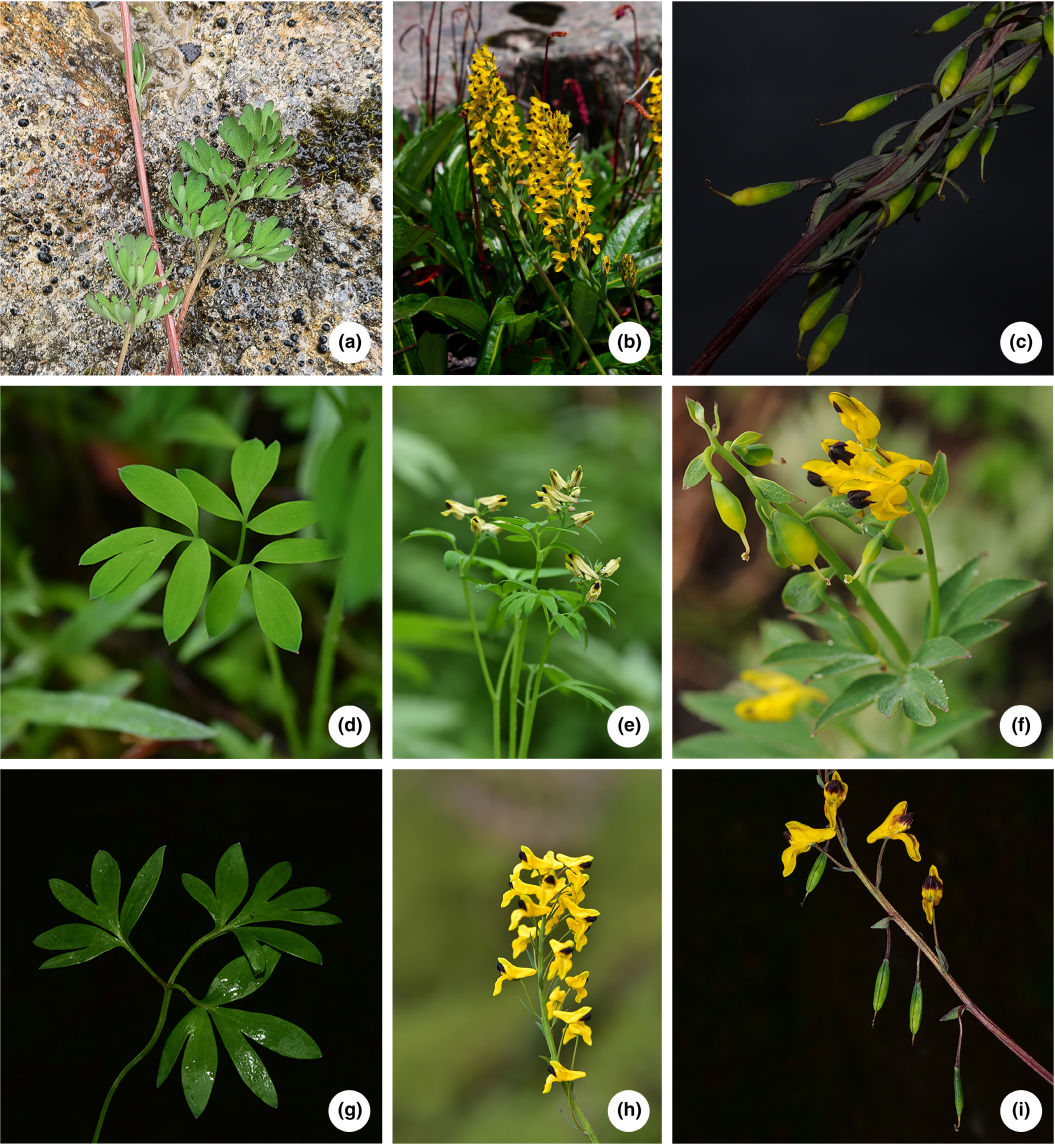
Morphological characters (showing radical leaves, inflorescences, and capsules from left to right) of *Corydalis sunhangii* J.T. Chen, T. Deng & M. Lidén (a–c), *C*. *kingdonis* (d–f), and *C. juncea* (g–i). (f) photographed by Bing Liu.

In sect. *Trachycarpae*, there are some species with rather smaller yellow flowers with inner petals with sharply contrasting black‐purple apex that are similar to *C. sunhangii*. We have selected *C. kingdonis* Airy Shaw for the morphological comparison, as it shares some general attributes with *C. sunhangii*, and has an adjacent distribution. It is fairly frequent around Duoxiongla on the south side of the Yarlung Zangbo River and at Galongla a bit further East. *C. juncea* Wallich is distributed in the central and eastern regions of the Himalayas, further away from this new species. Morphological comparisons of *C. sunhangii*, with the slightly similar taxa *C. kingdonis* and *C. juncea* are provided in Table 2 (Figure 1).

**TABLE 2:** Morphological comparisons of *Corydalis sunhangii*, *Corydalis kingdonis*, and *Corydalis juncea.*

Characters	*C. Sunhangii*	*C. Kingdonis*	*C. Juncea*
Radical leaves	Prominent, usually several; blade tripinnate	Insignificant, few; blade bi‐ to triternate	Insignificant, few, bi‐ to triternate
Cauline leaves	Usually one, usually situated in lower half of stem, much smaller than radical leaves, ovate, bipinnate, pinnate or palmatisect; ultimate lobes ca 1 mm broad	Usually two, situated in upper third of stem, like radical leaves but larger; ultimate lobes 2–4 mm broad	Usually one, sessile; blade lanceolate or linear‐lanceolate, entire
Inflorescences	Racemes 3–7 cm, densely 13–35‐flowered	Racemes 1.5–2.5 cm, rather lax, 4–11‐flowered	Raceme 5–20 cm, 10–30‐flowered
Flowers	Upper petal 12–13 mm, with broad rounded crest much overtopping apex; lower petal with shallowly saccate; nectary ca. 1/2–3/5 as long as spur	Upper petal 9–11 mm, with less than 1.0 mm crest not overtopping apex or without; lower petal with prominently and deeply saccate; nectary 2/3–4/5 as long as spur	Upper petal 10–15 mm, narrow crest decurrent on spur; spur cylindric, 5–7 mm; nectary ca. 1/2 as long as spur
Capsules	Oblong, with raised lines of dense papillae	Broadly obovoid, smooth	Oblong, smooth

### 3.2 Molecular phylogenetic analysis

The length of the concatenated 68 plastome CDS from 49 samples was 56,935 bp, of which 37,816 sites were constant (66.4%). Of these 68 plastome CDS, 60 CDS covered 49 individuals, five CDS covered 48, and three CDS covered 46.

**FIGURE 2:**
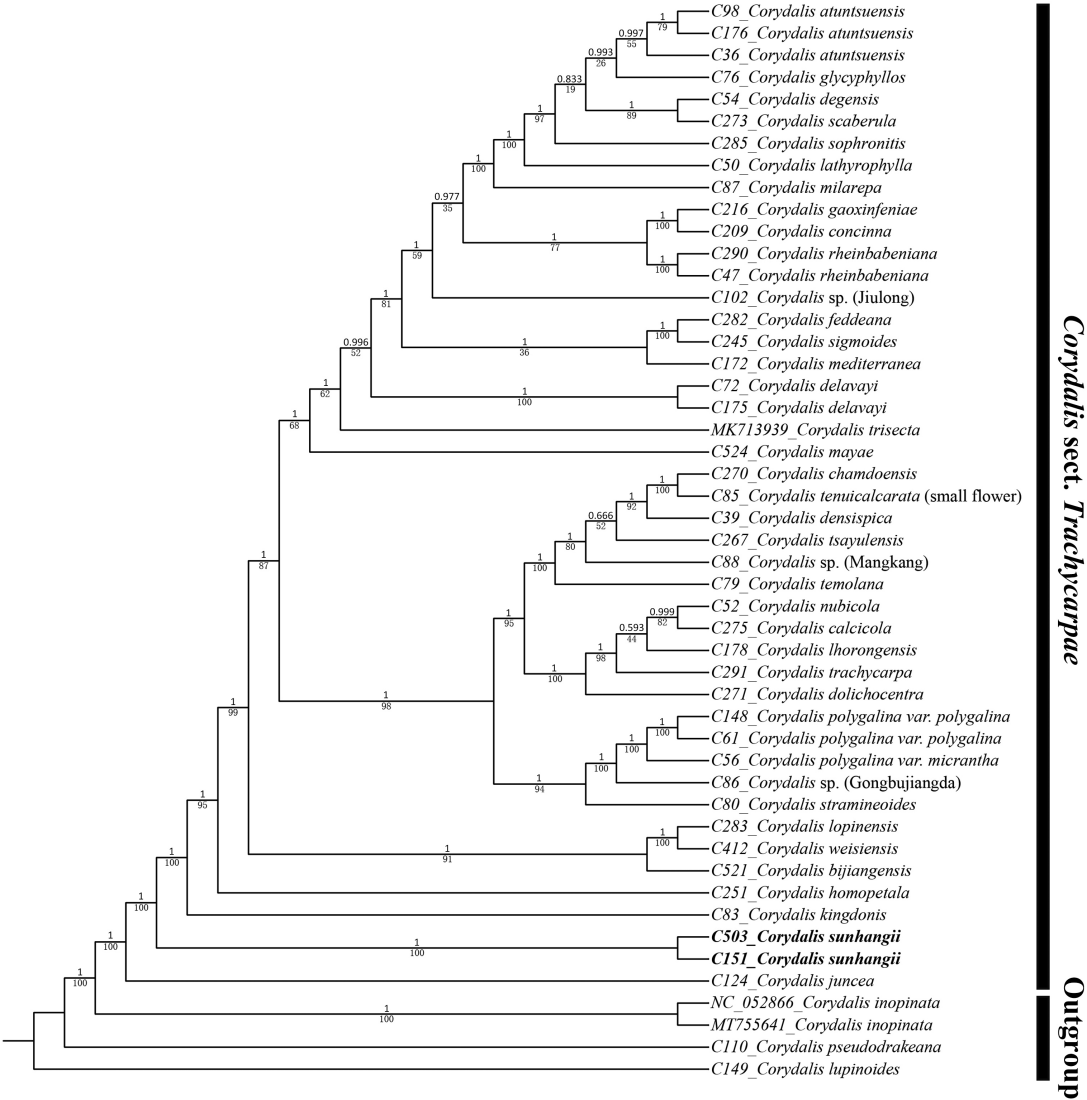
Phylogeny of *Corydalis* sect. *Trachycarpae* based on the combined 68 plastome CDS. Numbers above branches indicate Bayesian posterior probability (BIPP), numbers below branches represent maximum likelihood bootstrap support (MLBS). The new species is shown in bold.

Based on 68 shared protein‐coding plastid genes, BI and ML trees were reconstructed and their topologies are similar (Figure 2). The phylogenetic analyses show that all 45 samples of sect. *Trachycarpae* clustered into one clade with strong support (BIPP = 1, MLBS = 100%). Its sister group is *C*. sect. *Inopinatae* represented by *C. inopinata* Prain ex Fedde. Agreeing with previous analyses (Chen et al., 2023), *C. juncea* is sister to the rest of the sect. *Trachycarpae* in both trees. Both trees also show that our two accessions of the new species grouped together (BIPP = 1, MLBS = 100%) and form a sister group to the clade consisting of the remaining 42 samples of the section with high support (BIPP = 1, MLBS = 100%).

## 4 DISCUSSION

The monophyly of sect. *Trachycarpae* based on 68 plastome CDS from 45 plastomes is congruent with previous study (Chen et al., 2023) based on the combined 65 plastome CDS and 39 plastomes of sect. *Trachycarpae*. Here, the most comprehensive and robust phylogeny of sect. *Trachycarpae* was reconstructed to fully clarify relationships between this new species and other species in this section. In sect. *Trachycarpae*, early‐diverging lineages (*C. juncea*, *C. sunhangii*, *C. kingdonis*, *C. homopetala* Diels, *C. bijiangensis* C. Y. Wu et H. Chuang, *C. weisiensis* H. Chuang, *C. lopinensis* Franch.) have some similar morphological features, such as rather smaller yellow flowers with inner petals with sharply contrasting black‐purple apex, but the phylogenetic results suggest that these characters are plesiomorphic, that is, which do not indicate that these species sharing them are related. In the phylogeny of sect. *Trachycarpae*, this new species does not have a truly related sister species and adjacent to the new species are two isolated lineages (*C. juncea* and *C. kingdonis*). Morphologically, the new species is also similar to *C. juncea* and *C. kingdonis* in having small yellow flowers with inner petals with sharply contrasting black‐purple apex, but differs in having tripinnate radical leaves, different cauline leaves, densely 13–35‐flowered racemes, unique flowers and oblong capsules with raised lines of dense papillae. Considering the morphological data and phylogenetic results, we believe that this evidence satisfies the required diagnostic criteria to identify *C. sunhangii* as a new species.

**FIGURE 3:**
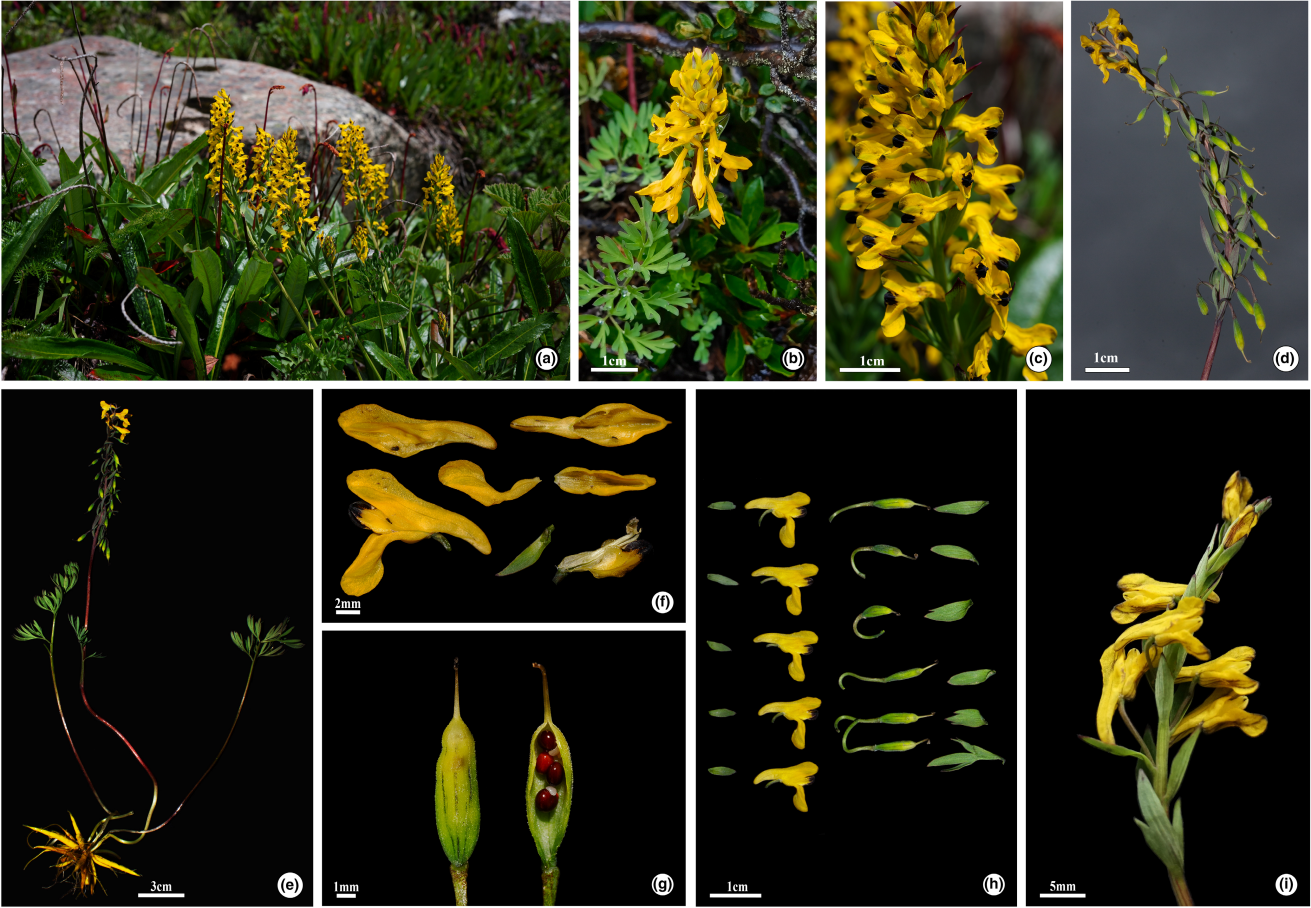
*Corydalis sunhangii* J.T. Chen, T. Deng & M. Lidén (a) habitat and plants; (b, c) racemes and radical leaves; (d) infructescence; (e) plant with roots; (f) floral parts; (g) capsule and seeds; (h) flowers and capsules; (i) plant with pale inner petals.

Additionally, we have discovered a unique individual (*QTP‐LuoJ‐1768*, Figure 3i) and its inner petals without sharply contrasting black‐purple apex, which is of great significance for understanding the morphological diversity.

**FIGURE 4:**
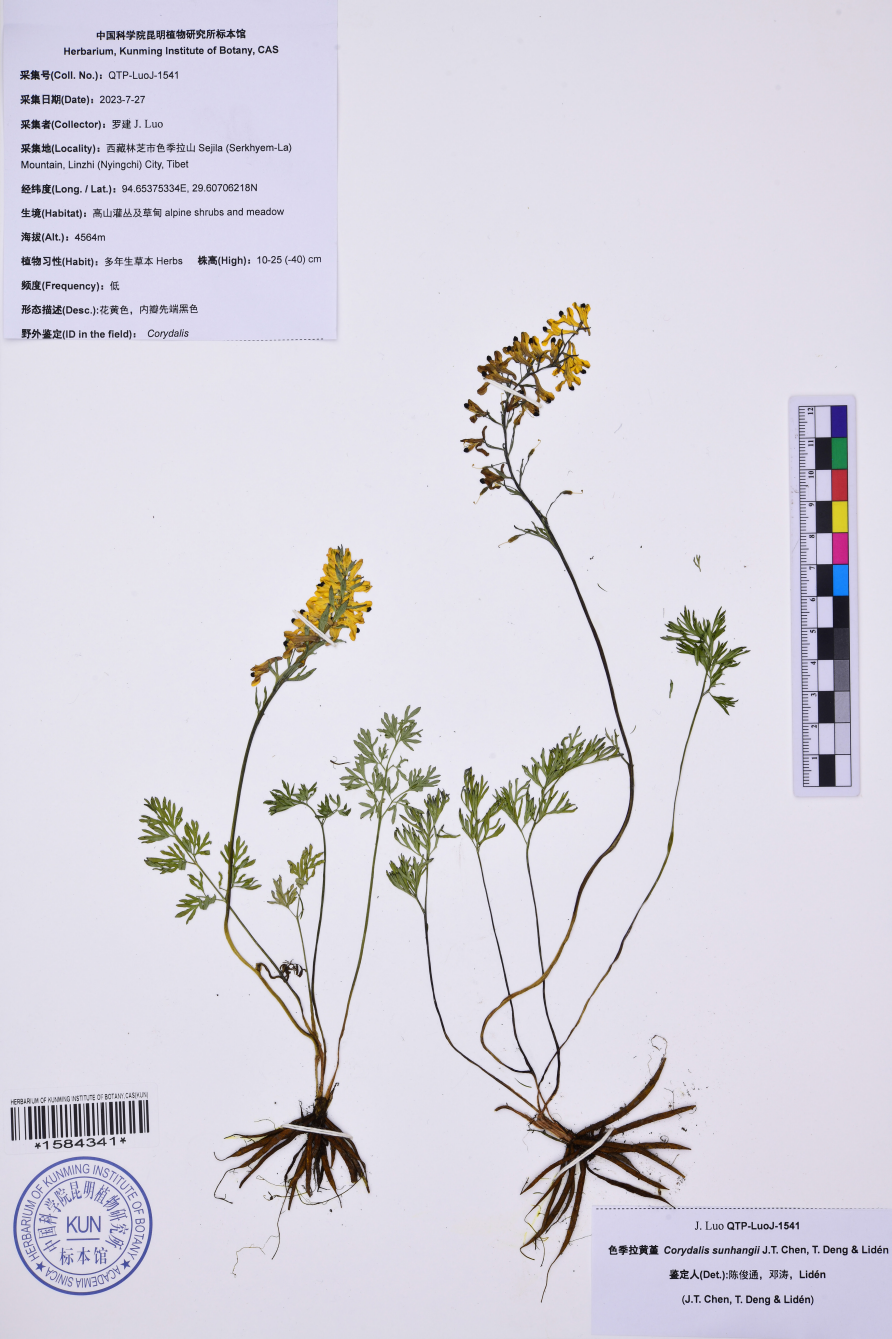
Photograph of the holotype of *Corydalis sunhangii* J.T. Chen, T. Deng & M. Lidén (KUN barcode 1,584,341).

Phylogeny based on plastid genome data has been used as important evidence to publish new species within *Corydalis* for the first time, which represents an important new trend in the development of taxonomy.

## 5 TAXONOMIC TREATMENT

***Corydalis sunhangii*** J.T. Chen, T. Deng & M. Lidén **sp. nov**.

Figures 3, 4, 5.

**FIGURE 5:**
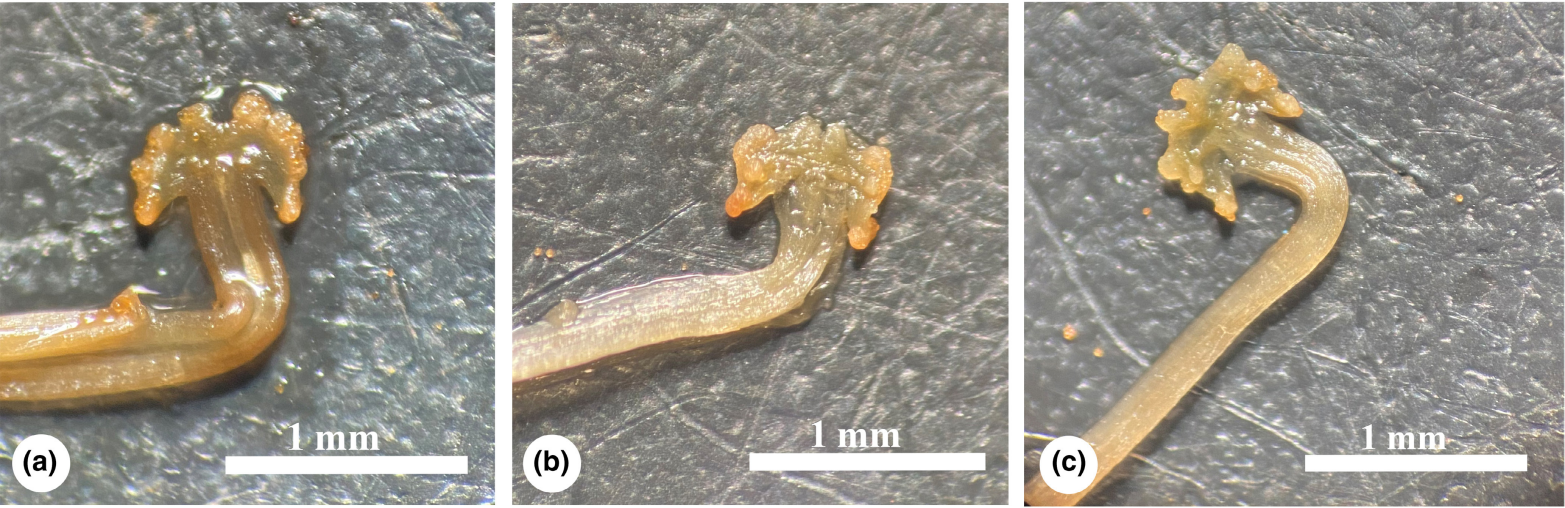
Three types of stigmas of *Corydalis sunhangii* J.T. Chen, T. Deng & M. Lidén.

**Type**. CHINA. Xizang: Nyingchi City, Bayi District, Sejila Mountain, 94.65375334 E, 29.60706218 N, elev. 4564 m, 27 Jul. 2023, *J. Luo QTP‐LuoJ‐1541* (holotype: KUN1584341!; isotype: KUN1584342!).

**Diagnosis.** *Corydalis sunhangii* resembles *C. kingdonis*, but differs by its radical leaves prominent, usually several, blade tripinnate (vs. insignificant, few, blade bi‐ to triternate); cauline leaf usually one, much smaller than radical leaves, usually situated in lower half of stem, ovate, bipinnate, pinnate, or palmatisect (vs. usually two, like radical leaves but larger, in upper third of stem, blade bi‐ to triternate); racemes 3–7 cm, densely 13–35‐flowered (vs. 1.5–2.5 cm, rather lax, 4–11‐flowered); claw of lower petal shallowly saccate (vs. very prominently and deeply saccate); capsule oblong, with raised lines of dense papillae (vs. broadly obovoid, smooth).

**Description.** Herbs, perennial, 10–25 (−40) cm tall, glabrous; stems and petioles of radical leaves attenuate to filiform underground base. Storage roots fasciculate, oblong to narrowly fusiform, (1–)2–4(−6) cm × 1–3 mm, fleshy, sessile. Stems 1–5, erect, simple, or rarely with a small axillary inflorescence from uppermost leaf. Radical leaves (1–) 2–4 (−6); petiole 3–10 (−19) cm long, with a wide semi‐hyaline slightly fleshy base; blade broadly ovate‐triangular, (1.8–) 2.5–5 × (1.5–) 2.0–4 cm, glaucous abaxially, green adaxially, tripinnate; ultimate segments narrowly lanceolate, 3–10 × 1.0–2.1 mm, acute or obtuse. Cauline leaves 0–2 (usually 1), smaller, ovate, 0.9–3.0 × 0.8–1.8 cm, biternate, bipinnate, pinnate, or palmatisect, petiole 3–10 (−25) mm long, slightly broadened at base. Racemes 3–7 cm long, narrow, densely 13–35‐flowered; most bracts narrowly lanceolate, entire, acuminate, 5–20 mm, or lowermost bract often pinnatifid or palmatisect, usually with purplish‐red apex. Pedicel 5–10 (−15) mm long, rather slender, arcuately reflexed in fruit. Sepals whitish, 0.6–1.0 × 0.3–0.6 mm, dentate. Corolla yellow, veins usually distinct; inner petals with sharply contrasting black‐purple apex, rarely just with four tiny spots at the top (Figure 3i); outer petals acute, with 1.5–2.0 mm broad rounded crest clearly overtopping apex; upper petal (8–) 12–13 mm long, crest slightly decurrent on spur; spur tapering to obtuse apex, 4–6 mm long, slightly downcurved; nectary ca. 1/2–3/5 as long as spur; lower petal, 6–9 mm long, claw shallowly saccate, limb ovate‐triangular, reflexed; inner petals 6–7 mm long. Stigma square, with 8, 10, or 12 papillae (Figure 5); geminate smaller papillae in lateral faces or absent; lateral edges papillae diffused or two fused into one broader papillae; larger papillae in basal corners. Capsule oblong, 6.0–8.5 × ca. 2 mm, with raised lines of dense papillae, 4–5‐seeded; style 3–4 mm. Seeds ca. 1.3 mm, smooth; elaiosome small, appressed.

**Phenology.** Flowering and fruiting from July to August.

**Etymology.** *Corydalis sunhangii* is named after Academician Hang Sun, who has made outstanding contributions to the flora of Pan‐Third Pole of the world. The Chinese name “色季拉黄堇” (sè jì lā huáng jǐn) refers to the Sejila Mountain where the species is found.

**Additional specimen examined (paratypes)—CHINA. Xizang.** Nyingchi City, Sejila Mountain, 94.651944 E, 29.610556 N, elev. 4550 m, 23 Jul. 2008, *J. Luo* & *S.L. Wang LiuJQ‐08XZ‐074* (KUN, HNWP); Sejila Mountain, elev. 4560 m, 11Aug. 2023, *Q. Liu*, *Q.L. Su*. and *Y.F. Li LQ‐SJL‐01*(KUN); Sejila Mountain, 94.653753241E, 29.60706218 N, elev. 4546 m, 19 Jul. 2023, *J. Luo QTP‐LuoJ‐1768*; Sejila Mountain, just W of Serkhyem La, 94.65056E, 29.61194 N, ca. 4500 m, stony ground close to running water, 18 Jul. 2019, J.C. Hao and M. Lidén s.n. (PE, UPS).

**Distribution and habitat.** The new species is known only from a small area in Nyingchi City, Xizang, China. It grows in the moist alpine shrubs and meadow at elevation of 4500–4600 m. Accompanying plants include *Polygonum griffithii* Hook. f. (Polygonaceae), *P. macrophyllum* var. *stenophyllum* (Meisn.) A.J.Li (Polygonaceae), *Rhododendron nivale* Hook. f. (Ericaceae), *Cassiope* sp. (Ericaceae), *Bergenia purpurascens* (Hook. f. et Thoms.) Engl. (Saxifragaceae), *Phlomis tibetica* C. Marquand & Airy Shaw (Lamiaceae), *Cassiope* sp. (Ericaceae), *Lilium nanum* Klotz. (Liliaceae), *Ligularia discoidea* S.W.Liu (Asteraceae), and *Rubus* sp. (Rosaceae).

**Conservation status.** *Corydalis sunghangii* is only known from Sejila Mountain, Nyingchi City. Further exploration is needed to assess its conservation status. Based on available data, the new species is assigned to the category “Data Deficient” (DD) of the International Union for Conservation of Nature (IUCN, 2022).

## AUTHOR CONTRIBUTIONS

**Jun‐Tong Chen:** Conceptualization (lead); data curation (equal); formal analysis (lead); writing – original draft (lead); writing – review and editing (equal). **Magnus Lidén:** Formal analysis (equal); writing – review and editing (equal). **Xian‐Han Huang:** Writing – review and editing (supporting). **Shun‐Quan Yang:** Writing – review and editing (supporting). **Xin‐Jian Zhang:** Writing – review and editing (supporting). **Qun Liu:** Investigation (equal). **Qi‐Lun Su:** Investigation (supporting). **Guo‐Jun Hua:** Investigation (supporting). **Jian Luo:** Investigation (lead). **Tao Deng:** Data curation (lead); funding acquisition (lead); supervision (lead); writing – review and editing (lead).

## FUNDING INFORMATION

The Biodiversity Survey and Assessment Project of the Ministry of Ecology and Environment, China (2019HJ2096001006), the Second Tibetan Plateau Scientific Expedition and Research (STEP) program (2019QZKK0502), National Natural Science Foundation of China (32300173 and 32322006), the Key Projects of the Joint Fund of the National Natural Science Foundation of China (U23A20149), Biological Resources Programme, Chinese Academy of Sciences (KFJ‐BRP‐017), and Survey of Wildlife Resources in Key Areas of Xizang (ZL202203601).

## CONFLICT OF INTEREST STATEMENT

The authors declare that there is no conflict of interest.

## Data Availability

The raw sequence data reported in this paper have been deposited in the Genome Sequence Archive (Genomics, Proteomics & Bioinformatics 2021) in National Genomics Data Center (Nucleic Acids Res 2022), China National Center for Bio information/Beijing Institute of Genomics, Chinese Academy of Sciences (GSA: CRA015506) that are publicly accessible at https://ngdc.cncb.ac.cn/gsa.
